# Clinical Analysis of Acute Organophosphorus Pesticide Poisoning and Successful Cardiopulmonary Resuscitation: A Case Series

**DOI:** 10.3389/fpubh.2022.866376

**Published:** 2022-05-27

**Authors:** Guangcai Yu, Yaqian Li, Tianzi Jian, Longke Shi, Siqi Cui, Liwen Zhao, Xiangdong Jian, Baotian Kan

**Affiliations:** ^1^Department of Poisoning and Occupational Diseases, Emergency Medicine, Cheeloo College of Medicine, Qilu Hospital of Shandong University, Shandong University, Jinan, China; ^2^Department of Digestive Internal Medicine, Cheeloo College of Medicine, Qilu Hospital of Shandong University, Shandong University, Jinan, China; ^3^School of Public Health, Cheeloo College of Medicine, Shandong University, Jinan, China; ^4^Department of Geriatric Medicine, School of Nursing, Cheeloo College of Medicine, Qilu Hospital of Shandong University, Shandong University, Jinan, China

**Keywords:** organophosphorus pesticide, poisoning, cardiac arrest, cardiopulmonary resuscitation, atropine

## Abstract

Acute organophosphorus pesticide poisoning (AOPP) with cardiac arrest has an extremely high mortality rate, and corresponding therapeutic strategies have rarely been reported. Therefore, this study aimed to explore the prognostic factors and effective treatments of AOPP-related cardiac arrest. This retrospective study was conducted in our department in the years 2018–2021. We conducted a descriptive analysis of the clinical manifestations, rescue strategies, and prognosis of patients with AOPP who had experienced cardiac arrest and successful cardiopulmonary resuscitation. This study included six cases of patients with AOPP in addition to cardiac arrest; in four cases, cardiac arrest occurred <12 h after ingestion, and in two, cardiac arrest occurred more than 48 h after ingestion. Five patients had not undergone hemoperfusion therapy before cardiac arrest, and all six were treated with atropine during cardiopulmonary resuscitation and subsequent pralidoxine. Four patients recovered and were discharged from the hospital, one died in our department, and one was transferred to a local hospital and died there 2 h later. The last two patients had severe pancreatic injuries and disseminated intravascular coagulation. This, along with their death, might have been related to their prognosis. Cardiac arrest can occur in patients with severe AOPP for whom antidote administration was insufficient or not timely. Application of atropine and pralidoxine in a timely manner after cardiac arrest following AOPP is the key to successful treatment. This study provides useful guidelines for the treatment of similar cases in the future.

## Introduction

Some organophosphorus pesticides (OPs) are extremely poisonous and cause rapid intoxication-induced death with minimal ingestion or exposure. Acute organophosphorus pesticide poisoning (AOPP) is a major life-threatening toxic disease in the rural areas of developing countries (1, 2). In China, AOPP cases comprise nearly 50% of all poisoning cases, with case fatality rates of 3–40%, comprising over 80% of all poisoning deaths (3, 4). OPs cause damage to multiple organs through cholinergic and non-cholinergic effects (5). Common symptoms of AOPP include central nervous system and neuromuscular complications, with cardiopulmonary arrest being the most serious complication and often having a very poor prognosis (6, 7). However, AOPP-related cardiac arrest is rarely reported. In this study, we analyzed the clinical data of patients with AOPP who experienced cardiopulmonary arrest and successful cardiopulmonary resuscitation (CPR) on-site and summarized the clinical characteristics and prognostic factors of the aforementioned disease.

## Materials and Methods

### Study Participants

Six patients with AOPP who suffered a cardiac and/or respiratory arrest and were successfully resuscitated on-site were selected as research participants. These patients had been admitted to the Department of Poisoning and Occupational Diseases, QILU Hospital of Shandong University (Jinan City, China) between January 1, 2018 and December 31, 2021.

### Inclusion and Exclusion Criteria

The inclusion criteria included a patient age of ≥18 years, patients who ingested OPs orally, a time from ingestion to admission to our department of <48 h, and meeting the following diagnostic and exclusion criteria. The diagnostic criteria were a clear history of taking poison, a distinct garlic or petroleum odor after ingestion, a reduced acetylcholinesterase level, a cholinergic crisis, and a positive trial of atropine. Patients with previous heart disease or other diseases, such gastroenteritis, myasthenia gravis, Guillain-Barré syndrome, botulism, mushroom toxicity, and nicotine toxicity, were excluded.

### Treatment Plan

When patients were transferred to our department, blood and urine routine examinations, along with coagulation function, liver function, renal function, creatine kinase-MB, amylase, lipase, blood glucose, blood lipids, electrolyte, and cholinesterase tests, were conducted. Moreover, other related examinations were obtained. The main conventional treatment drugs included penehyclidine hydrochloride injection (1 mg, twice daily), atropine (1 mg, every 6 h and adjusted as necessary), pralidoxime iodide (2.0 g, twice daily), betamethasone (8 mg, once daily), pantoprazole (40 mg, twice daily), reduced glutathione (1.8 g, once daily), alanyl glutamine (20 g, once daily), torsemide (20 g, twice daily), nalmefene (0.1 mg, twice daily), and fat emulsion, amino acid (8), and glucose (1%) injections (1,920 mL, once daily). Hemoperfusion was administered twice in the first 24 h of admission and subsequently once daily for a total of four times, which was a treatment plan that we called the “2-1-1 plan.” The treatment plan was adjusted appropriately based on disease progression. When a cholinergic crisis occurred, atropine was given in a timely manner, and when cardiac and/or respiratory arrest occurred, CPR was immediately performed, and atropine was administered simultaneously. All patients after CPR were timely treated with pralidoxime. We also administered smectite powder and injected activated carbon with mannitol into the patients' stomach through a gastric tube for gastrointestinal decontamination.

### Data Collection and Analysis

The patient data described in this paper were obtained from the Department of Poisoning and Occupational Diseases, QILU Hospital of Shandong University (Jinan City, China). We conducted a descriptive analysis of the whole medical record related to this study. Data on the sex, age, type of poison, medical history, main treatment, and condition changes (e.g., prehospital treatment, treatment with CPR, and disease progression) of each patient were obtained. Continuous variables are presented as means ± standard deviations, and categorical variables are presented as counts or actual numerical values.

### Registration and Ethics

This study was approved by the Ethics Committee of the Shandong University QILU Hospital (Jinan City), and written informed consent was obtained from the families of patients.

## Results

Six patients with AOPP were included in the study. There was one man and five women who were aged 49–66 years, with an average age of 56.8 ± 6.0 years. This research involved four cases of dichlorvos poisoning, one of chlorpyrifos poisoning, and one of dimethoate poisoning. All six patients developed cardiac arrest and were administered atropine by intravenous injection during CPR. Five patients received endotracheal intubation and mechanical ventilation during CPR, and one received mechanical ventilation before cardiac arrest because of respiratory failure. Five patients had not undergone hemoperfusion before the onset of cardiac and/or respiratory arrest. After treatment, four patients recovered and were discharged, and two died. The main clinical data of patients are shown in Table 1.

**Table 1:** Clinical data of six patients with acute organophosphorus pesticide poisoning.

**Patient**	**Sex (F/M)**	**Age (y)**	**Pesticide**	**Prehospital gastric lavage (Y/N)**	**Hemoperfusion before cardiac arrest (Y/N)**	**Endotracheal intubation and mechanical ventilation**	**Dosage of atropine during CPR**	**Time to enter our department after ingestion**	**Time of cardiac arrest**	**Prognosis**
Patient 1	F	66	Chlorpyrifos	Y	N	During CPR	2 mg (one time)	1.5 h	Occurred 2.5 h after ingestion	Recovered
Patient 2	F	56	Dichlorvos	Y	N	During CPR	5 mg (1 mg, 2 mg, 2 mg)	14 h	When transferred to the local hospital	Death
Patient 3	F	50	Dichlorvos	Y	N	10 h before cardiac arrest	3 mg (2 mg, 1 mg)	12 h	Occurred 15 min after being transferred to our department	Recovered
Patient 4	F	62	Dichlorvos	N	N	During CPR	10 mg (5 mg, 5 mg)	10 h	When transferred to the local emergency department	Death
Patient 5	M	49	Dimethoate	Y	N	During CPR	2 mg (1 mg, 1 mg)	2 d	Occurred 5 d after ingestion	Recovered
Patient 6	F	58	Dichlorvos	Y	Y	During CPR	5 mg (2 mg, 2 mg, 1 mg)	2 h	Occurred 58 h after ingestion	Recovered

*F, female; M, male; Y, yes; N, no; and CPR, cardiopulmonary resuscitation*.

Four patients who had lower cholinesterase levels on admission had cardiac arrest <12 h after ingestion, that is, when they were immediately transferred to a local hospital or our hospital. Two patients who were administered reduced atropine and/or pralidoxime iodide had cardiac arrests on the fifth day and third day after ingestion, respectively. Both of the patients who died had severe pancreatic injury (amylase and/or lipase levels were three times higher than baseline values) and abnormal coagulation on admission. The patients' main laboratory tests results are shown in Table 2.

**Table 2:** Main laboratory test results of six patients upon admission to our department.

**Inspection item**	**Normal range**	**Patient 1**	**Patient 2**	**Patient 3**	**Patient 4**	**Patient 5**	**Patient 6**
CHE (IU/L)	4,650–10,440 IU/L	<200	<200	<200	<200	497.	201
pH	7.35–7.45	7.36	7.21	7.27	7.02	7.35	7.26
Lac (mmol/L)	0.5–2.2 mmol/L	2.9	1.2	1.4	1.1	6.7	1.0
CK (IU/L)	30–135 IU/L	NA	224	88	NA	158	NA
ALT (IU/L)	9–52 IU/L	15	120	27	91	18	17
AST (IU/L)	14–36 IU/L	25	159	32	136	24	34
CK-MB (ng/ml)	0.3–4.0 ng/mL	1.6	18.7	6.5	48.2	6.0	9.2
Amylase (IU/L)	30–110 IU/L	NA	1,611	254	2,509	67	349
Lipase (IU/L)	23–300 IU/L	NA	3,134	99.76	326	37	218
PT (s)	11–14.5 s	14.4	17.4	25.8	25.5	10.4	13.3
PT-INR	0.8–1.2	1.11	1.46	1.23	2.32	0.9	1.01
APTT (s)	28–45 s	28.4	37.5	>180	73.3	24.8	39.1
D-dimer (μg/mL)	0–0.5 μg/mL	5.09	11.33	2.3	>20	0.35	0.65
Fib (g/L)	2–4 g/L	2.72	1.76	3.99	0.60	3.13	3.93
FDP (μg/L)	0–5 μg/L	19.29	42.05	7.52	>150	NA	2.22

*CHE, cholinesterase; pH, pondus hydrogenii; Lac, lactic acid; CK, creatine kinase; ALT, alanine aminotransferase; AST, aspartate aminotransferase; CK-MB, creatine kinase-MB; PT, prothrombin time; PT-INR, prothrombin time normalized ratio; APTT, activated partial thromboplastin time; Fib, plasma fibrinogen; FDP, fibrinogen degradation product; NA, not available*.

## Discussion

In this study, we analyzed the clinical data of patients with AOPP who experienced cardiopulmonary arrest and successful CPR on-site. We found that cardiac arrest occurred in patients with severe AOPP for whom antidote application was insufficient or untimely. Therefore, we assumed that cardiac arrest was related to the toxic effect of OPs and administration of an insufficient amount of a specific antidote.

AOPP is a toxic disease having a main symptom of a cholinergic crisis, leading to the phosphorylation of serine residues in the active site of acetylcholine esterase (AChE) and gradual inhibition of AChE (9). The reactivation of AChE *in vivo* is key to the successful treatment of AOPP. The main treatment includes atropine, oxime drugs, the removal of toxins *in vivo*, and symptomatic supportive treatment (10–12). The determination of AChE activity can be used as an important index for the diagnosis, grading, and judgment of AOPP. OPs can inhibit acetylcholinesterase and butyrylcholinesterase, although the inhibition of butyrylcholinesterase does not produce clinical symptoms for the most part (13). In China, nearly all clinical hospitals only detect serum levels of cholinesterase, that is, butyrylcholinesterase; therefore, determining a response to AOPP therapy based on butyrylcholinesterase is not completely reliable (14, 15). The lack of a specific antidote or an impertinent rapid diagnosis and disease evaluation leads to improper treatment (16). Atropine and oxime-type antidotes should be applied in a timely manner for AOPP. If the cholinergic crisis induced by AOPP cannot be treated in a timely manner, it is highly likely to progress to cardiopulmonary arrest (17).

In patients with severe AOPP, OPs may cause central apnea or hypopnea (8), and a cholinergic crisis can lead to increased airway secretion, acute cholinergic respiratory failure, and respiratory arrest (18). OPs can also inhibit heart function and cause bradycardia and cardiac arrest. Hypoxemia, electrolyte derangements, and acidosis are major predisposing factors for cardiac arrest (19), and close monitoring and airway management are essential for prevention. The initial dose of atropine for adults is 2 to 5 mg intravenously, and if the patient does not respond to treatment, the dose must be doubled every 3 to 5 min until respiratory secretions have cleared and there is no bronchoconstriction. In severe cases, treatment may require continuous infusion over several days; at first, the toxicants in the gastrointestinal tract are not completely removed, although atropine temporarily alleviates symptoms of cholinergic crisis. However, the amount of antidote in the body remains relatively insufficient, and a cholinergic crisis can easily occur. Thus, once a patient experiences cardiac and/or respiratory arrest, atropine and oxime-type antidotes should be administered simultaneously with CPR (18, 19). In this study, patients who had successful CPR had the common characteristic of the application of atropine and establishment of advanced airway management during on-site resuscitation.

Atropine only works on muscarinic receptors, and pralidoxime works by reactivating the phosphorylated AChE by binding to OPs. The detoxification of oxime, as a specific antidote for AOPP, has saved many lives through early appropriate intervention (20–22). The standard of care includes a bolus of at least 30 mg/kg over 30 min. After the bolus, a continuous infusion of at least 8 mg/kg/h should be initiated and may be needed for several days. However, for the detoxification of oxime to work, it need to be given within 48 h of poisoning. Animal studies have shown that pralidoxime can contribute to the successful resuscitation of cardiac arrest in organophosphate-induced pig models (23). The outlook for most patients is excellent, although cardiac arrest occurred in some severe cases (3). However, owing to the limitation in the acetylcholinesterase structure-based design of oxime antidotes for AOPP, some detoxification effects are limited for some OPs (24, 25). Gastrointestinal decontamination and hemoperfusion (26) can remove OPs in the gastrointestinal tract and blood, respectively. Therefore, the timely and complete removal of toxins is another essential treatment for AOPP. This can also reduce the incidence of poisoning rebound and reduce the incidence of cardiopulmonary arrest.

The suitable dose of atropine for AOPP-related cardiac arrest has not been reported yet. In a previous edition of the American Heart Association Guidelines for Cardiopulmonary Resuscitation and Emergency Cardiovascular Care (2020), atropine was not involved in CPR (27), and in a consensus of clinical experts on the diagnosis and treatment of AOPP (2016) in China (4), it had suggested that atropine should be actively administered for AOPP-related cardiac arrest; however, the exact dose was not discussed. In this paper, we observed that the practical dose of atropine during CPR was between 2 and 10 mg for patients undergoing an AOPP-induced cardiac arrest. Due to the limitation of only six patients in this study, the optimal dose of antidotes is not entirely clear, and further research is warranted.

Patients with severe AOPP must be timely evaluated after receiving treatment, and antidotes, such as atropine and pralidoxime, should be administered as soon as possible. Hemoperfusion, gastrointestinal decontamination, and respiratory support treatment should be administered when necessary. For patients with respiratory and cardiac arrest, atropine and pralidoxime are of great importance during CPR. We believe that our findings could potentially provide guidelines for the treatment of AOPP-related cardiopulmonary arrest. Further studies should be conducted to determine the dose and administration time of specific antidotes during CPR.

## Data Availability Statement

The original contributions presented in the study are included in the article/supplementary material, further inquiries can be directed to the corresponding author/s.

## Ethics Statement

This study was approved by the Ethics Committee of Shandong University Qilu Hospital (Jinan City). Written informed consent was obtained from the patients' families. The patients/participants provided their written informed consent to participate in this study. Written informed consent was obtained from the individual(s) for the publication of any potentially identifiable images or data included in this article.

## Author Contributions

GY conceived the study, drafted the manuscript, and all authors contributed substantially to its revision. GY, YL, TJ, and LZ supervised the conduct of the paper and data collection. GY, XJ, YL, LS, SC, and LZ provided statistical advice on the study design and analyzed the data. BK and XJ chaired the data oversight committee. GY and YL take responsibility for the paper as a whole. All authors contributed to the article and approved the submitted version.

## Funding

This project was funded as a scientific research project of the Qilu Hospital, Shandong University (Project Number: KYLL-2019-296).

## Conflict of Interest

The authors declare that the research was conducted in the absence of any commercial or financial relationships that could be construed as a potential conflict of interest.

## Publisher's Note

All claims expressed in this article are solely those of the authors and do not necessarily represent those of their affiliated organizations, or those of the publisher, the editors and the reviewers. Any product that may be evaluated in this article, or claim that may be made by its manufacturer, is not guaranteed or endorsed by the publisher.
